# Pediatric and Juvenile Strabismus Surgery Under General Anesthesia: Functional Outcomes and Safety

**DOI:** 10.3390/jcm14041076

**Published:** 2025-02-08

**Authors:** Jakob Briem, Sandra Rezar-Dreindl, Lorenz Wassermann, Katharina Eibenberger, Franz Pusch, Ursula Schmidt-Erfurth, Eva Stifter

**Affiliations:** 1 Department of Ophthalmology and Optometry, Medical University of Vienna, 1090 Vienna, Austria; 2 Department of Anesthesiology, Medical University of Vienna, 1090 Vienna, Austria

**Keywords:** esotropia, exotropia, strabismus surgery, pediatric ophthalmology

## Abstract

**Background/Objectives:** The aim of this paper was to evaluate the safety of surgical intervention using anesthesia and ophthalmological parameters in pediatric strabismus patients. The design involved retrospective case series. **Methods:** The setting was the Department of Ophthalmology, Medical University Vienna, Austria. Participants: In total, 208 children aged 0–18 years who underwent strabismus surgery due to exotropia or esotropia between 2013 and 2020 were included. Main outcomes and measures: Information regarding the duration of surgery, intra- and postoperative complications, the postoperative angle of deviation (AoD), and functional outcomes (visual acuity, stereovision) were analyzed. **Results:** The mean age at the time of surgery was 6.0 ± 4.1 years (range 0.6–18.0). The mean anesthesia time among all patients was 75.9 ± 19.3 min. The mean surgery and anesthesia time did not differ between the age groups. Longer anesthesia durations and surgery durations did not have a significant effect on the occurrence of intraoperation complications (*p* = 0.610 and *p* = 0.190, respectively). Intraoperative complications were recorded in 53% (most frequent triggering of oculocardiac reflex (OCR)) of the patients, and postoperative complications in 22% (the most frequent were postoperative nausea and vomiting and pain). An OCR was triggered more often in children older than 6 years than in younger children (*p* = 0.016). The mean angle of deviation was significantly reduced from preoperative to postoperative measurements. Preoperative stereovision tests were positive in 35% of the patients and increased to over 80% postoperatively. **Conclusions:** Strabismus surgery performed under general anesthesia in children aged 0 to 18 years is safe with regard to both surgical and anesthetic complications. A significant decrease in the angle of deviation and high rate of stereovision could be achieved with a low rate of re-treatments. However, the retrospective design, absence of standardized documentation, and limited sample size may affect the consistency and comparability of this study’s findings.

## 1. Introduction

Strabismus surgery is one of the most common pediatric ophthalmic procedures [1]. The incidence of strabismus in Europe is about 2.5%, and the prevalence varies between 3 and 7% [2,3,4]. In pediatric patients, surgical procedures for strabismus are usually performed under general anesthesia with the intention of restoring the alignment of the eyes to maintain good visual acuity, and to improve depth perception and binocular vision [5]. The psychosocial consequences of untreated strabismus in children must always be considered as well [6]. Occlusion therapy, pharmacological treatments, and the use of spectacles and/or prisms are among the non-surgical options used to treat strabismus [7]. To reduce the risk of amblyopia, it is essential to treat pediatric strabismus early [8].

Since regional ophthalmic block or topical anesthesia requires a cooperative adult patient, general anesthesia is the anesthetic management of choice in pediatric patients undergoing strabismus surgery. The surgery demands a lot from anesthesiologists because traction on the extraocular muscles can cause oculocardiac and oculorespiratory reflexes, which may result in bradycardia, dysrhythmias, hypoxemia, and hypercapnia during surgery [1]. Postoperatively, many patients experience nausea and vomiting and/or pain, which causes emotional distress in pediatric patients and also delays in recovery room discharge [9,10].

Numerous surgical techniques are used for correcting strabismus in children. Typically, the eye muscles are re-positioned by resection, recession, or folding of the muscles. Severe intraoperative complications are rare but do happen occasionally. The reported rate of severe complications is about 1% and includes scleral penetrations and perforations, raised intraocular pressure, snapped/lost muscle, periorbital infections, and scleritis [11,12]. Recurrence of strabismus is common, especially in children. Over- and under-correction are relatively frequent, and vertical misalignments can also develop throughout life. Reoperations are therefore sometimes necessary [8,13].

Given that many forms of pediatric strabismus require surgical intervention, it is of great importance to assess the safety and efficacy of such procedures. Understanding potential complications during anesthesia and throughout the intra- and postoperative phase is critical for improving treatment outcomes in this patient population. The existing literature on strabismus lacks comprehensive studies that consider both anesthesiological and ophthalmological parameters simultaneously. Research addressing the relationship between anesthesia and surgical durations with factors such as patient age, the number of operated muscles, and other clinical variables is limited. Additionally, there is insufficient evidence of the association between surgery or anesthesia duration and the occurrence of intraoperative complications, highlighting the necessity for further investigation in these areas. Therefore, the primary goal of this study was to assess the safety of strabismus surgery under general anesthesia in children aged 0 to 18 years. This will be accomplished by analyzing the incidence of intra- and postoperative complications and assessing differences in complication rates and severity based on patient age, anesthesia, surgery duration, and the type of strabismus surgery performed.

## 2. Materials and Methods

This retrospective case series was performed at the Department of Ophthalmology, Medical University of Vienna, Austria. This study adhered to the tenets of the Declaration of Helsinki and was approved by the local ethics committee of the Medical University of Vienna (EK:1212/2021). The ethics committee did not ask for informed consent as this was a retrospective chart review.

In total, 208 children with congenital esotropia or exotropia were included in the analysis. The medical history, surgery reports, and anesthesia- and recovery-room protocols of children aged 0–18 years with esotropia or exotropia that underwent first-time unilateral or bilateral recession or folding surgery of extraocular muscles between November 2013 and December 2020 were evaluated. Children with strabismus associated with systemic diseases (e.g., Down’s syndrome, Graves’ disease, Duane syndrome, Moebius syndrome), with strabismus due to brain tumors or neurological diseases (e.g., nerve palsies), and patients with a history of penetrating or perforating eye trauma or strabismus caused by severe head injury (e.g., orbital wall fracture) were excluded from this study. Patients with solely vertical strabismus were excluded because this study aimed to focus on horizontal strabismus surgeries. Patients who had undergone strabismus surgery before were excluded to minimize confounding factors from prior operative treatment.

The outcome measures were divided into functional postoperative outcomes, anesthesia parameters, safety (intra- and postoperative complications), and re-treatment. Descriptive data of the patients including sex and age (at initial presentation and at surgery) and the time from the first visit until surgery were collected. Anesthesia outcomes, composed of duration of surgery and anesthesia (measured in minutes, min)—that is, total time spent in the operation room, total anesthesia time (intubation to extubation), extubation time (time between the end of surgery and extubation), and time spent in the recovery room—were recorded. Further anesthesia parameters included the type of airway management used (endotracheal tube, laryngeal mask, or other), the type of anesthetic agent administered, and the type of additional intraoperative medication given (analgesics, antiemetics, anticholinergics, muscle relaxants, or others). Safety was defined as the incidence of intraoperative and postoperative complications resulting from strabismus surgery or anesthesia. The types of intraoperative complications, such as surgical and anesthetic complications (oculocardiac reflex, incidences of oxygen decreases below 90%, intraoperative CO_2_ increases, arrythmia/tachycardia, scleral penetrations and perforations, snapped/lost muscle, retinal detachment, and pulled-in-two syndrome), as well as the type and number of postoperative complications (pain, postoperative nausea, and vomiting, among others), were examined by reviewing the anesthesia protocols, surgery reports, recovery room protocols, and patient records.

In addition, data were collected on the necessity of re-treatment for strabismus and the type of re-treatment (surgical, non-surgical). The postoperative outcome was assessed at two time intervals, 1–3 months and 3–6 months after surgery. Since children of different ages demonstrate developmental and physiological differences that may influence anesthetic requirements or may lead to surgical challenges and higher complication rates, three age groups were formed to compare the collected data: under 3 years old, 3 to 6 years old, and over 6 years old at the time of surgery. To determine whether more extensive surgeries might require longer anesthesia times and carry a higher risk of complications, additional groups were created based on the number of muscles operated on (2, 3, 4, or 5 muscles).

### Statistical Analysis and Outcome Measures

Statistical analysis was performed using descriptive statistics for all variables and data frequency. Nominal data are given as numbers or percentages, whereas normally distributed continuous data are given as the mean ± standard deviation (SD). The analysis aimed to evaluate the safety and general functional outcome of pediatric patients undergoing strabismus surgery. Nominal variables were compared using a X^2^-test or a Fisher exact test if the minimum expected frequency could not be reached in 1 cell of the 4-field chart. A Levane test was performed to test the homogeneity of variances for the continuous variables, which included total surgery time, total time spent in the operation room, total anesthesia time, extubation time, and time spent in the recovery room. Following this, a two-way multivariate analysis of variance (MANOVA) was used to investigate the relationship between the independent variables (age at time of surgery, amount of muscles operated on) and the dependent continuous variables mentioned above. A logistic regression analysis was performed to investigate the relationship between anesthesia duration/surgery duration and the occurrence of intraoperative complications. Furthermore, X^2^-tests were conducted to assess the impact of patient age and the number of operated muscles on the occurrence of intraoperative complications. Statistical analyses were conducted with SPSS. *p*-values < 0.05 were considered statistically significant.

## 3. Results

This study included 416 eyes of 208 children, with a mean age at first presentation of 4.1 ± 4.1 years (ranging from 0.03 to 17.7). The mean age at the time of strabismus surgery was 6.0 ± 4.0 years (ranging from 0.6 to 18.0). Esotropia was diagnosed in 74.5% (155 out to 208) of the patients, while exotropia was diagnosed in 25.5% (53 out of 208) of the patients. More than a third of the patients (37.5%) presented with additional vertical eye deviation. Table 1 provides general information about the study population.

### 3.1. Anesthesia

The average total anesthesia time among all patients was 75.9 ± 19.3 min (range: 42–153 min). A two-way MANOVA was conducted to examine the impact of age at the time of surgery and the number of muscles operated on on the duration of anesthesia. The results indicated that age had no significant effect on the total time spent in the operating room, the total anesthesia time, or the total surgery time (*p* > 0.05). The only factor that influenced the duration was the number of muscles operated on. Table 2 and Table 3 provide detailed information regarding the anesthesia parameters, airway management, anesthetic agents, and pre- and postoperative medications.

### 3.2. Intraoperative Complications

The strabismus operations performed on our patient group were safe with regard to anesthesia and ophthalmological parameters. There were no serious, life-threating complications in any of the patients. During the surgeries, 106 patients (53%, out of a total of 202) experienced intraoperative complications. Six patients had incomplete anesthesia protocols and were therefore excluded from the analysis. The most commonly reported complication was the triggering of the oculocardiac reflex (OCR), which occurred in 100 patients (49%). In three patients (1%), SpO2 dropped below 90%, while one patient (0.5%) experienced tachycardia. There were no cases of scleral penetration/perforation, snapped/lost muscle, retinal detachment, or pulled-in-two syndrome in our patient population. There were no statistically significant differences observed in the occurrence of intraoperative complications based on either the number of muscles operated or the age of the patients (*p* = 0.344, X^2^-test and *p* = 0.066, X^2^-test, respectively). Furthermore, the type of airway management showed no statistically significant effect on the occurrence of intraoperative complications (*p* = 0.156, X^2^-test).

A logistic regression analysis was performed to investigate the relationship between anesthesia duration and the occurrence of intraoperative complications. The results indicate that longer anesthesia duration does not have a significant effect on the occurrence of intraoperative complications (*p* = 0.610; ß = −0.0037; OR: 1.00, [95% CI: 0.98, 1.01]). Furthermore, longer surgery duration also showed no significant effect on the occurrence of intraoperative complications (*p* = 0.190; ß = 0.0011; OR: 1.01, [95% CI: 0.99, 1.03]). OCR was defined as a sudden 10% drop from baseline heart rate resulting from traction on an eye muscle or pressure on the eyeball. There was a high incidence of OCR in our patient population. Nearly half of the children (49%; n = 100/202) experienced at least one instance of a 10% drop in heart rate during the surgery. No cases of asystole were reported. The occurrence of OCR was significantly higher in children over the age of six compared to younger patients (*p* = 0.016, X^2^-test). No anticholinergic premedication was administered as standard in our hospital. The incidence of OCR was found to be significantly higher in the initial 10 min of surgery (72%) compared to the remainder of the procedure (28%). Detailed information on the intraoperative complications can be found in Table 4 and Table 5.

### 3.3. Postoperative Complications

Postoperatively, a total of 46 patients (22%) experienced 53 complications. Nineteen children (9%) experienced postoperative pain with a numeric-rating-scale/visual analog scale (NRS/VAS) greater than 3, requiring analgesic intervention. PONV was observed in 13 patients (6%), without a statistically significant difference in occurrence among the age groups (*p* = 0.513, X^2^-test).

In terms of ophthalmological complications, the most frequently observed postoperative finding was bacterial conjunctivitis, affecting eight patients (4%), followed by Tenon’s prolapse in four patients (2%), conjunctival cysts in two patients (1%), and suture granuloma in two patients (1%).

Re-treatment of strabismus was required in 22 out of 208 patients (11%). The methods of re-treatment included re-surgery in 20 patients (10%), the use of prism glasses in four patients (2%), and orthoptic exercises in the clinic for one patient (0.5%). Short-term surgical re-treatments (first 12 months post-operation) were necessary for four children. The main reasons for surgical re-treatment were postoperative consecutive strabismus divergens/convergens (25%), an increase in vertical deviation (15%), strabismus in the previously unoperated eye (15%), and diplopia (15%).

### 3.4. Mean Angle of Deviation

The mean preoperative angle of deviation (AoD) was 32 ± 10 prism diopters (pdp) at distance and 34 ± 12 pdp when near. One to three months after surgery, the mean deviation decreased significantly to 7 ± 7 pdp at distance and 9 ± 8 pdp when near. The surgical success rate (defined as AoD < 20 pdp postoperatively) was 92.64% and 89.6% of patients for distance and near deviations, respectively. At 3–6 months postoperatively, the success rates were 89.29% and 83.92% for distance and near deviation.

The vertical deviation at distance improved from 9 ± 6 pdp preoperatively to 3 ± 5 pdp 1–3 months postoperatively. Near vertical deviation improved from 10 ± 6 pdp preoperatively to 4 ± 5 pdp in the first 3 postoperative months, and slightly worsened to 6 ± 7 pdp at months 3–6.

### 3.5. Functional Outcome

Preoperatively, data from Lang or Bagolini tests were available for 91 patients. Of these, 32 patients (35%) had a positive result in at least one of the administered tests, whereas 59 patients (65%) demonstrated a negative result. Postoperatively, the proportion of patients achieving a positive result in at least one stereovision test increased to over 80% (114 patients).

There was minimal change in visual acuity from the preoperative to postoperative period. Specifically, for the right eye, the mean logMAR visual acuity was 0.17 ± 0.25 before the procedure and 0.17 ± 0.25 after the procedure. Similarly, for the left eye, the preoperative mean logMAR visual acuity was 0.17 ± 0.24 and 0.16 ± 0.22 postoperatively.

## 4. Discussion

In this study, we were able to demonstrate the safety of both combined strabismus surgery performed on the horizontal eye muscles and strabismus surgery performed on the straight and oblique eye muscles. Although intra- and postoperative complications were relatively common, no serious life-threatening complications occurred. We were able to achieve a significant reduction in the angle of deviation with a low rate of re-operation. Additionally, we observed a significant increase in positive stereovision testing and minimal changes in visual acuity, indicating improved functional outcomes.

The analysis of general anesthesia data provided interesting results. The age at time of surgery did not have an impact on the total time spent in the operating room, the total anesthesia time, or the total surgery time. The only factor that influenced the durations was the number of muscles operated on.

Intraoperative complications were relatively common in our study population. Serious intraoperative complications such as slipped or lost muscle, retinal detachment, pulled-in-two syndrome, or scleral/globe perforation were not observed in our population compared to studies conducted by other authors [11,12,14,15]. The most common intraoperative complication in our study, the triggering of the oculocardiac reflex, occurred in about half of the patients at any time within the operation. These rates are consistent with the literature, where the incidence of OCR varies widely, ranging from 10 to 90%, depending on the definition used [16,17,18,19,20,21,22]. We defined the OCR as a sudden drop of at least 10% from baseline heart rate after traction on an eye muscle/pressure on the eyeball. Because the exact timepoint of the first traction on an eye muscle was not recorded in the anesthesia protocols, we chose the first 10 min after the documented first incision of surgery as the realistic period in which the first traction of a muscle must have occurred. Sudden intraoperative drops in heart rate above 10% were also considered an OCR, although traction on a muscle could not be reliably verified as the cause of the OCR in these cases. The reflex was triggered significantly more often in children over 6 years of age than in younger children. Due to the retrospective nature of this study and the resulting lack of standardized documentation of intraoperative complications in the anesthesia protocols, underreporting or overreporting of complications, such as the OCR, is possible, thereby limiting the reliability and interpretability of the data.

There is an ongoing debate in the literature about what the best option is for reducing the incidence and severity of OCR [18,23,24]. If the reflex is triggered intraoperatively and severe drops in heart rate occur, we recommend immediate cessation of muscle traction and the intraoperative administration of anticholinergic drugs to restore a normal cardiac rhythm. Other authors showed similar results with the use of these drugs in their studies [18,22]. All other intraoperative complications in our study were rare. There were no surgeon-initiated complications.

We observed only minor postoperative complications among our patients, with PONV and pain having the most common incidence rates of 6% and 9%, respectively. Studies have shown that the incidence of postoperative nausea and vomiting in pediatric patients is approximately twice that of adults, ranging from 10 to 80% [20,23,25]. Our study reported a lower incidence of PONV compared to other authors, but under-reporting may have occurred due to the retrospective nature of this study. At our hospital, we frequently administer a combination of dexamethasone and a serotonin-receptor antagonist to patients who are at an elevated risk of experiencing PONV, a dual medication therapy that other authors have also reported as the most effective antiemetic treatment [1,26,27]. In our study population, nearly 10% of the children required analgesic treatment for postoperative pain. Intravenous NSAIDS are the most suitable for this purpose as they produce similar postoperative pain scores as opioids, thereby reducing opioid consumption [27,28].

After surgery, almost every patient showed signs of local inflammation like chemosis or conjunctival injection; however, treatment was not necessary. Complications like conjunctival cysts or suture granulomas were less common, but most resolved without treatment. Some minor localized infections like bacterial conjunctivitis occurred in our patients, but severe infections like endophthalmitis or orbital cellulitis were not observed [15].

We also focused on the general functional outcome of the patients in this study. The key factor in achieving a good functional outcome and a desirable cosmetic result is a low postoperative deviation. Within the first three months following the surgery, we were able to observe a significant reduction in the angle of deviation, both near and at distance. However, during later postoperative follow-ups, some patients experienced a slight worsening of the deviation, indicating that the final postoperative results can only be seen after several months, if not years. Other studies also showed that over time, the number of patients with worsening deviation continues to increase [29,30,31]. Compared to other authors, we report a lower rate of re-surgery; however, due to the variable follow-up studies, these are not directly comparable with each other [30,31,32,33,34]. Louwagie et al. conducted a long-term study with a median follow-up period of 11.9 years, which revealed that the probability of requiring a second strabismus operation continuously increased over the years [32]. Therefore, more studies are needed to examine the long-term outcomes of patients undergoing strabismus surgery.

With regard to binocular vision, we observed a significant increase in positive test results among our patients from pre- to postoperative assessments, with almost 80% of patients displaying at least one positive result in a binocular test during their last follow-up visit. However, other studies have reported significantly lower numbers of patients with positive stereovision [32,35].

More prospective long-term studies examining the development of binocular function after strabismus surgery using a standardized postoperative protocol are needed.

## 5. Conclusions

Despite the retrospective nature of this study, it has been shown that strabismus surgery in children is very safe with regard to both anesthesia and ophthalmological complications. There were no serious complications that occurred either during or after the surgery. A longer duration of surgery and anesthesia had no effect on the occurrence of intraoperative complications. PONV and postoperative pain were among the most frequent postoperative complications, but could be managed very well. Several limitations must be acknowledged in this study. The retrospective design may have resulted in the underreporting of complications due to the absence of a standardized framework for documenting intraoperative and postoperative events and complications, which restricts the consistency and comparability of the data. Another limitation of this study is the small sample size. These limitations should be considered when interpreting the findings and highlight the importance of conducting future prospective studies utilizing standardized protocols and lager datasets to ensure more robust and generalizable findings.

## Figures and Tables

**Table 1:** Baseline characteristics of the total study population.

Sex (Male–Female)	113:95
**Age, years (mean ± standard deviation)**	6.0 ± 4.0
Age 0–3 years (n (%))	56 (26.9)
Age 3–6 years (n (%))	67 (32.2)
Age >6 years (n (%))	85 (40.9)
**Type of strabismus (number (%))**	
Strabismus convergens	155 (74.5)
Strabismus divergens	53 (25.5)
Strabismus horizontalis et verticalis	78 (37.5)
Strabismus convergens et verticalis	61 (29.3)
Strabismus divergens et verticalis	17 (8.2)
**Number of eyes operated (number (%))**	
one	132 (63.5)
two	76 (36.5)
**Number of muscles operated (number (%))**	
two muscles	111 (53.4)
three muscles	50 (24.0)
four muscles	35 (17.0)
five muscles	12 (5.8)

**Table 2:** Detailed information on the anesthesia times in strabismus surgery among the subgroups depending on age and number of muscles operated on.

	**Amount of Muscles Operated**	**Total Study Population**	**Age < 3 Years**	**Age 3–6 Years**	**Age > 6 Years**
Total time in operating room (min)	2	77.9 ± 15.9 (111)	80.6 ± 21.2 (22)	79.3 ± 14.6 (27)	76.3 ± 14.3 (62)
	3	92.1 ± 15.2 (50)	89.2 ± 14.9 (13)	91.5 ± 16.7 (23)	95.8 ± 13.1 (14)
	4	106.3 ± 20.6 (35)	107.5 ± 18.4 (15)	111.1 ± 24.1 (12)	96.6 ± 17.8 (8)
	5	125.4 ± 11.2 (12)	124.6 ± 11.4 (6)	127.4 ± 12.9 (6)	120.0 (1)
Total anesthesia time (min)	2	66.1 ± 13.8 (109)	66.5 ± 16.1 (22)	67.2 ± 14.8 (26)	65.5 ± 12.7 (61)
	3	79.2 ± 14.1 (50)	73.4 ± 14.8 (13)	81.9 ± 13.9 (23)	80.3 ± 13.1 (14)
	4	91.9 ± 19.4 (33)	89.8 ± 18.1 (13)	97.8 ± 23.4 (12)	86.3 ± 13.9 (8)
	5	107.3 ± 12.5 (12)	108.5 ± 15.0 (6)	106.6 ± 11.9 (5)	103.0 (1)
Total surgery time (min)	2	37.4 ± 10.9 (111)	36.0 ± 10.7 (22)	39.2 ± 10.9 (27)	37.2 ± 11.0 (62)
	3	49.3 ± 11.7 (50)	47.2 ± 12.4 (13)	50.1 ± 13.0 (23)	49.9 ± 8.9 (14)
	4	61.4 ± 16.4 (35)	59.7 ± 15.4 (15)	63.8 ± 20.0 (12)	60.9 ± 13.6 (8)
	5	70.9 ± 15.4 (12)	64.0 ± 19.0 (6)	77.0 ± 7.3 (5)	82.0 (1)
Extubation time (min)	2	15.4 ± 8.2 (109)	16.6 ± 10.7 (22)	15.4 ± 9.4 (26)	14.9 ± 6.6 (61)
	3	16.3 ± 7.5 (50)	11.8 ± 5.5 (13)	18.4 ± 5.9 (23)	16.9 ± 9.9 (14)
	4	15.5 ± 7.2 (32)	17.6 ± 8.7 (13)	14.9 ± 6.4 (11)	12.8 ± 4.7 (8)
	5	19.3 ± 11.8 (12)	18.8 ± 13.4 (6)	21.8 ± 11.5 (5)	10.0 (1)
Time at the recovery-room (min)	2	103.3 ± 35.9 (101)	111.2 ± 43.3 (21)	117.1 ± 42.5 (25)	94.1 ± 26.2 (55)
	3	109.0 ± 44.5 (46)	101.2 ± 27.8 (11)	120.9 ± 57.3 (22)	95.5 ± 23.0 (13)
	4	111.5 ± 30.7 (31)	112.2 ± 30.8 (13)	117.5 ± 39.8 (10)	102.8 ± 14.8 (8)
	5	114.3 ± 29.1 (7)	110.0 ± 16.8 (4)	97.5 ± 31.8 (2)	165.0 (1)

All data: mean ± SD (n).

**Table 3:** Detailed information on airway management for pre- and intraoperative medication.

Airway Management		
Endotracheal tube	40 (19.2)	
Laryngeal Mask	164 (78.8)	
**Preoperative medication**		
Midazolam	43 (20.7)	
No premedication	161 (77.4)	
**Intraoperative medication**		
Intravenous anesthetic		Mask induction using sevoflurane ^a^
propofol only	1 (0.5)	0 (0.0)
fentanyl only	9 (4.3)	4 (44.4)
propofol + fentanyl	174 (83.7)	79 (45.4)
propofol + remifentanil	10 (4.8)	1 (10.0)
propofol + fentanyl + remifentanil	8 (3.8)	1 (12.5)
propofol + fentanyl + ketamine	1 (0.5)	1 (100.0)
fentanyl + ketamine	1 (0.5)	0 (0.0)
Anticholinergic medication		
glycopyrrolate only	48 (23.1)	
atropine only	12 (5.8)	
glycopyrrolate + atropine	2 (1)	
Muscle relaxant		
rucoronium	7 (3.4)	
no muscle relaxant	197 (94.7)	
Analgesia		
paracetamol	166 (80.0)	
ibuprofen	4 (2.0)	
metamizole	6 (3.0)	
diclofenac	2 (1.0)	
piritramid	1 (0.5)	
Antiemetic medication		
dexamethasone only	28 (13.5)	
ondansetron only	22 (10.6)	
dexamethasone + ondansetron	41 (19.7)	
no antiemetic medication	113 (54.3)	

All data: n (%); n = 204. The data from four anesthesia protocols were excluded due to incomplete information. ^a^ percentage of patients in the corresponding intravenous anesthetic group.

**Table 4:** Information on the intraoperative complications in the total study population and among the subgroups depending on the amount of muscles operated on.

	Total Study Population	2-Muscle Surgery	3-Muscle Surgery	4-Muscle Surgery	5-Muscle Surgery	*p* Value
	n = 202	n = 109	n = 48	n = 33	n = 12	
Number of intraoperative complications	106 (52.5)	58 (53.2)	22 (45.8)	17 (51.5)	9 (75.0)	0.344 ^a^
Oculocardiac reflex	100 (49.5)	55 (50.5)	21 (43.8)	16 (48.5)	8 (66.7)	
Tachycardia	1 (0.5)	1 (0.9)	0	0	0	
Oxygen decrease below 90%	3 (1.5)	2 (1.8)	1 (2.1)	0	0	
CO_2_ increase	2 (1.0)	0	0	1(3.0)	1 (8.3)	
Scleral penetration/perforation	0	0	0	0	0	
Snapped/lost muscle	0	0	0	0	0	
Retinal detachment	0	0	0	0	0	
Pulled-in-two syndrome	0	0	0	0	0	

All data: n (%). ^a^ χ^2^ test.

**Table 5:** Information on the intraoperative complications in the total study population and among the subgroups depending on age.

	Total Study Population	Age < 3 Year	Age 3–6 Years	Age > 6 Years	*p* Value
	n = 202	n = 54	n = 65	n = 83	
Number of intraoperative complications	106 (52.5)	24 (44.4)	31 (47.7)	52 (62.6)	0.066 ^a^
Oculocardiac reflex	100 (49.5)	21 (38.9)	28 (43.1)	51 (61.4)	
Tachycardia	1 (0.5)	1 (1.8)	0	0	
Oxygen decrease below 90%	3 (1.5)	1 (1.8)	1 (1.5)	1 (1.2)	
CO_2_ increase	2 (1.0)	1 (1.8)	1 (1.5)	0	
Scleral penetration/perforation	0	0	0	0	
Snapped/lost muscle	0	0	0	0	
Retinal detachment	0	0	0	0	
Pulled-in-two syndrome	0	0	0	0	

All data: n (%). ^a^ χ^2^ test.

## Data Availability

The data are available upon reasonable request.
